# Characterization of Acute Poisoning in Hospitalized Children in Southwest China

**DOI:** 10.3389/fped.2021.727900

**Published:** 2021-12-10

**Authors:** Zhu Li, Li Xiao, Lin Yang, Shaojun Li, Liping Tan

**Affiliations:** ^1^Ministry of Education Key Laboratory of Child Development and Disorders, Chongqing Key Laboratory of Pediatrics, National Clinical Research Center for Child Health and Disorders, Children's Medical Big Data Intelligent Application Chongqing University Engineering Research Center, Department of Emergency, Children's Hospital of Chongqing Medical University, Chongqing, China; ^2^Ministry of Education Key Laboratory of Child Development and Disorders, Chongqing Key Laboratory of Pediatrics, National Clinical Research Center for Child Health and Disorders, Children's Medical Big Data Intelligent Application Chongqing University Engineering Research Center, Department of Hematology and Oncology, Children's Hospital of Chongqing Medical University, Chongqing, China

**Keywords:** children, acute poisoning, pesticides, drugs, suicide

## Abstract

**Objective:** Acute poisoning in children is characterized by regional differences. This study described the basic situation of childhood poisoning in southwest China based on hospitalized cases.

**Data and Methods:** A total of 1,076 acute poisoning cases among hospitalized children admitted to Children's Hospital of Chongqing Medical University from January 2012 to December 2020 were included in this study. Clinical data such as gender, age, living environment, poisonous substance, and cause of poisoning were statistically described. Trends of length of hospital stay, population distribution, poisonous substances, and causes of acute poisoning in the hospitalized children were compared over time.

**Results:** The cohort comprised 588 males and 488 females; 811 cases lived in rural areas and the rest resided in urban areas. Most cases were between early childhood and school age. Poisoning usually occurred at home (973 cases, 90.4%). Pesticides (381 cases, 35.4%) and drugs (275 cases, 25.6%) were the two most common poisonous substances. Two main causes of poisoning were accidental taking (755 cases, 70.2%) and suicide (177 cases, 16.4%). The results of univariate analysis of suicide showed significant correlations among gender, place of residence, age, poisonous substance, and place of suicide (*P* < 0.001), while living environment (town), age (adolescence), and poisonous substance (pesticide, drug) were independent risk factors (*P* < 0.05). There was no significant change in the length of hospital stay for poisoning over time. The overall number of hospitalizations presented a decreasing trend, while the number of urban children gradually increased. The proportion of adolescent poisoned children and suicidal children increased in the last 3 years.

**Conclusion:** Optimizing the package and distribution channels of pesticides and drugs, raising safety awareness of children to avoid accidental injuries, and paying attention to children's mental health are measures that are necessary to prevent poisoning in children.

## Introduction

Poisoning has been an important global public health issue, and acute poisoning is an important cause of unintentional injuries in children (1). Acute poisoning refers to a series of pathophysiological changes and relevant clinical manifestations in a short period of time after exposures to toxic substances or toxic doses of drugs. The consequences of acute poisoning are complex and change rapidly. Multiple organ dysfunction or failure can be observed in serious poisoning cases, which can be life-threatening (2). The World Health Organization (WHO) states that poisoning is one of the top five causes of death from unintentional injuries in children (3), and surveys from China also show that poisoning is one of the leading causes of death in Chinese children, ranking as high as the 3rd cause of accidental death (4). Despite numerous educational and public health campaigns aimed at avoiding childhood poisoning, poisoning remains the most common medical emergency among children. Children are more susceptible to serious injuries relative to adults due to their immature psychological and physical systems, lack of hazard awareness, and poor safety awareness and defense against poisons.

Acute childhood poisoning varies greatly from a global epidemiological perspective, with geography being a major factor for poisonous substances and risk factors (5). Some national epidemiological studies have been established in western countries, such as the European Association of Poisons Centers and Clinical Toxicologists (EAPCCT), Extracorporeal Treatments in Poisoning Work group (EXTRIP), National Poison Data System (NPDS), and the American Academy of Clinical Toxicology (AACT). Unfortunately, there remains a lack of large-scale, multicenter epidemiological data about the incidence of poisoning in Chinese populations, especially among children.

Children's Hospital of Chongqing Medical University is one of five national regional children's medical centers in China and the largest national tertiary class A children's specialty hospital in southwest China. Therefore, the acute poisoning cases from hospitalized children in this hospital are regionally representative. In this study, we included hospitalized children diagnosed with acute poisoning at the Children's Hospital of Chongqing Medical University from January 2012 to December 2020. We retrospectively analyzed their clinical characteristics, including length of hospital stay, poisoning population, poisoned substances, and causes of poisoning over time. This study is of significance for the prevention and control of acute poisoning in children.

## Data and Methods

### Selection of Research Subjects

Medical information of 1,101 hospitalized children diagnosed with acute poisoning from January 2012 to December 2020 at the Children's Hospital of Chongqing Medical University was collected from the hospital information management system. Twenty-five cases were excluded based on the following criteria: lack of clinical information, milder outpatient acute poisoning, non-acute poisoning, infectious poisoning, and poisonous insect and snake bites. All included subjects were <18 years old.

### Research Method Design

The clinical data of 1,076 hospitalized children with acute poisoning were statistically described, including basic characteristics (age, gender, underlying diseases, distribution of residential area), place of poisoning, poisonous substances, and causes of poisoning. Univariate analysis and multivariate logistic regression analysis were used to analyze the poisoning characteristics of suicidal children. Trends in length of hospital stay, population distribution, poisonous substances, and causes of poisoning in hospitalized children with acute poisoning over the past 9 years were further compared.

### Statistical Analysis

Data were analyzed using SPSS 22.0. Categorical variables are presented as cases/%. Ordinal and nominal categorical data were evaluated using rank sum test and Chi-square test, respectively. Enumeration variables are expressed as mean ± standard deviation. An independent sample *t*-test was used to compare two groups. One-way ANOVA was applied to compare differences among multiple groups assuming normal distribution, or the Kruskal-Wallis test was used when necessary. Bonferroni *post-hoc* test was performed for pair-wise comparisons. Univariate analysis was used for correlation factor analysis, followed by multi-factor logistic regression analysis. All results were considered statistically different at *P* < 0.05.

## Results

### Basic Characteristics of Hospitalized Children With Acute Poisoning

The basic characteristics of children enrolled in this study are summarized in Table 1. There were 588 males and 488 females, and the gender ratio was 1.2:1. The top three age groups of poisoning were early childhood, preschool, and school ages (291, 312, and 273 cases, respectively). Forty-eight cases were characterized by past history of underlying diseases, including 11 cases of depression and epilepsy. The number of children in rural areas was much higher than in urban areas.

**Table 1:** Basic characteristics of hospitalized children with acute poisoning.

**Name**			**Number of cases**	**Percentage (%)**
**Gender**				
	Male		588	54.6
	Female		488	45.4
Age^*^				
	Pre-adolescence	Total	919	85.4
		Neonatal period	9	0.8
		Infancy	34	3.2
		Early childhood	291	27
		Pre-school	312	29
		School age	273	25.4
	Adolescence		157	14.6
**Underlying diseases**				
	None	Total	1,028	95.5
		Neither the child nor the family	1,004	93.3
		Family members on medication	24	2.2
	Yes	Total	48	4.4
		Patient depression	24	2.2
		Patient's other illnesses (epilepsy, cerebral palsy, etc.)	24	2.2
**Place of residence**				
		Rural	811	75.4
		Urban	265	24.6

* *age groups: neonatal period (<28 days old), infancy (28 days old to <1 year old), early childhood (1 to <3 years old), pre-school (3 to <7 years old), school age (7 to <13 years old), and adolescence (13 to 18 years old)*.

### Place of Poisoning, Poisonous Substances, and Cause of Poisoning

Poisoning accidents mainly occurred at home (90.4%), followed by other places, including schools (9 cases), health care facilities (10 cases), and outdoors (18 cases). The three most common poisonous substances were pesticides, drugs, and rat poisons. Paraquat poisoning (236 cases) was the most prominent, accounting for 21.9% of pesticide poisonings. Rodenticides were dominated by anticoagulants (dallon and bromadiolone). The most common cause of poisoning was accidental ingestion of poisonous substances. According to the characteristics of children, accidental ingestion was classified as self-inflicted and accidentally fed by others. Suicide was another cause of poisoning (Table 2).

**Table 2:** Distribution of poisonous substances, poisoning causes, and poisoning locations.

**Name**			**Number of cases**	**Percentage (%)**
**Poisonous substance**				
	Pesticides	Total	381	35.4
		Organophosphorus	79	7.3
		Diquat	30	2.8
		Paraquat	236	21.9
		Others (etofenprox, methomyl, etc.)	36	3.3
	Drugs		275	25.6
	Rat poison	Total	164	15.2
		Poisonous rat poison	39	3.6
		Bromadiolone/Dalon	81	7.5
		Other rat poison	44	4.1
	Other	Total	257	23.8
		Other (CO, alcohol, nitrite, daily chemical products, etc.)	134	12.4
		Poisonous mushroom	123	11.4
**Cause of poisoning**				
	Non-self	Total	899	83.6
		Accidentally taken by oneself	755	70.2
		Others accidentally fed	48	4.5
		Other (poisoning, contact, etc.)	96	8.9
	Suicide		177	16.4
**Place of poisoning**				
	Unknown		66	6.1
	At home		973	90.4
	Not at home (school, medical		37	3.4
	facility, outdoors, etc.)			

Pesticides and rodenticides were significantly higher in rural areas compared to urban areas (*P* < 0.001). The highest rates of pesticide poisoning were observed in rural areas, while the highest rates of drug poisoning were found in urban areas (Table 3).

**Table 3:** Distribution of poisonous substances in urban and rural areas (cases/%).

**Name**	**Pesticide**	**Drug**	**Rat poison**	**Other**
Rural	350/43.20%	142/17.4%	136/16.8%	184/22.7%
Urban	31/11.7%	133/50.2%	28/10.6%	73/27.5%
*x* ^2^	534.178	0.589	142.244	5.429
*p*	<0.001	0.443	<0.001	0.02

Univariate analysis of poisoning characteristics of the 177 suicidal children showed that gender, residential area, age, previous history, and poisonous substances were all significantly associated with suicide (*P* < 0.001). The rate of suicide was higher in females than in males and increased with age. The rate of suicide was higher among those living in urban areas. Cases with basic/underlying diseases were characterized by a higher rate of suicide. Drugs and pesticides were the most prevalent poisonous substances (Table 4).

**Table 4:** Univariate analysis of suicide.

**Factor**		**Non-suicide**	**Suicide**	***X*^2^/*Z***	***p***
**Gender**					
	Male	517	71	18.056	<0.001
	Female	382	106		
**Place of residence**					
	Rural	697	114	13.721	<0.001
	Urban	202	63		
**Age**					
	Pre-adolescence	820	36	456.684	<0.001
	Adolescence	79	141		
**Underlying diseases**					
	None	877	151	52.002	<0.001
	Yes	22	26		
**Poisoned substance**					
	Other (Poisonous mushroom, alcohol, CO, etc.)	246	11	61.996	<0.001
	Pesticides	295	86		
	Rat poison	152	12		
	Drugs	206	68		

By performing the binary multifactorial logistic regression analysis of the poisoning characteristics of children who committed suicide, we found that residential areas (urban), age (adolescence), and poisonous substances (pesticides, drugs) were independent risk factors for suicide (Table 5).

**Table 5:** Multifactorial analysis of suicide.

**Factor**	**B**	**S.E**.	**Wald**	**df**	***p***	**Exp(B)**	**95% lower limit**	**95% upper limit**
Male	0.108	0.24	0.204	1	0.652	1.114	0.697	1.782
Urban	0.698	0.297	5.502	1	0.019	2.009	1.122	3.599
With underlying diseases	0.75	0.531	1.991	1	0.158	2.116	0.747	5.994
Adolescence	3.731	0.244	232.97	1	<0.001	41.724	25.841	67.369
Poisonous substance-others			38.535	3	0			
Substance-pesticides	2.115	0.398	28.188	1	<0.001	8.29	3.797	18.098
Substance-rat poison	0.678	0.496	1.868	1	0.172	1.97	0.745	5.21
Substance-drugs	1.986	0.426	21.703	1	<0.001	7.289	3.16	16.81

### Trends in Poisoning Characteristics From 2012 to 2020

#### Hospitalization Days

The number of hospitalization days is summarized in Table 6. The length of hospitalization stay from 2012 to 2020 was significantly different (*P* < 0.05).

**Table 6:** Hospitalization days between 2012 and 2020.

**Year**	Median (IQR^*^)	***X* ^2^**	***p***
2012	5 (6)	71.209	0.028
2013	5 (6)		
2014	5 (7)		
2015	5 (5)		
2016	5 (8)		
2017	6 (7)		
2018	5 (6)		
2019	5(6)		
2020	4 (4)		

* *IQR, Interquartile spacing*.

#### Number of Cases

**Figure 1:**
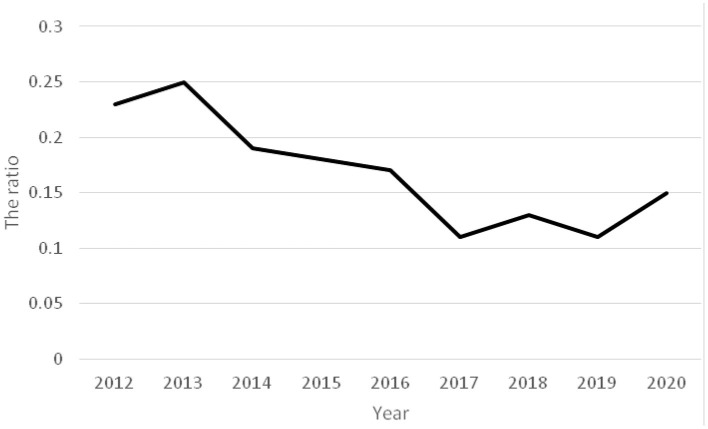
Time-varying curve of the ratio of poisoning in children to total hospital admissions.

**Figure 2:**
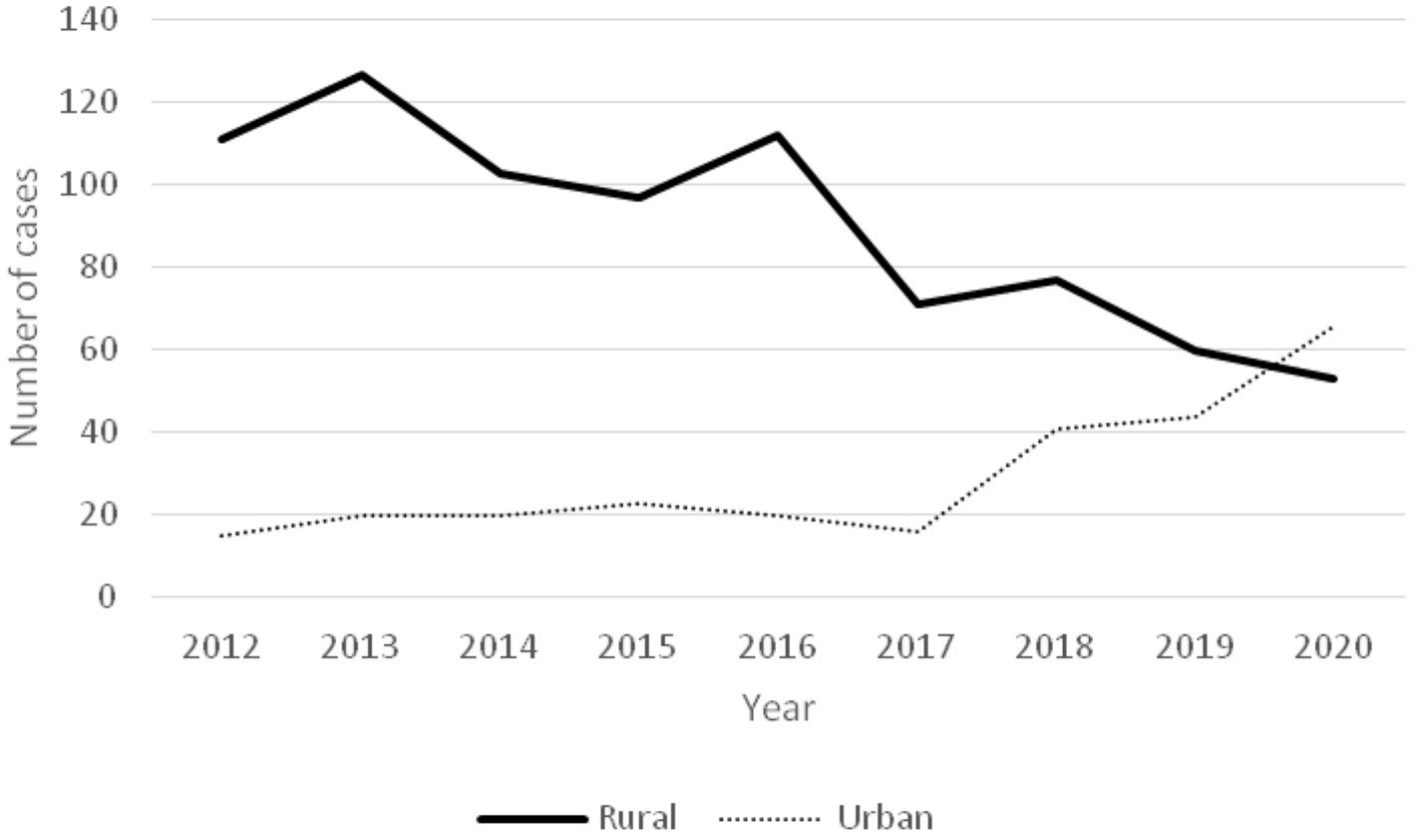
Time-varying curve of the number of poisoning in children in rural and urban areas.

The ratios of acute poisoning cases to total hospitalized children showed a decreasing trend from 2012 to 2020 (Figure 1). A gradual increase in the number of poisoning in children from urban areas was observed, while the number of cases in rural area decreased (Figure 2).

#### Age Group Distribution

**Figure 3:**
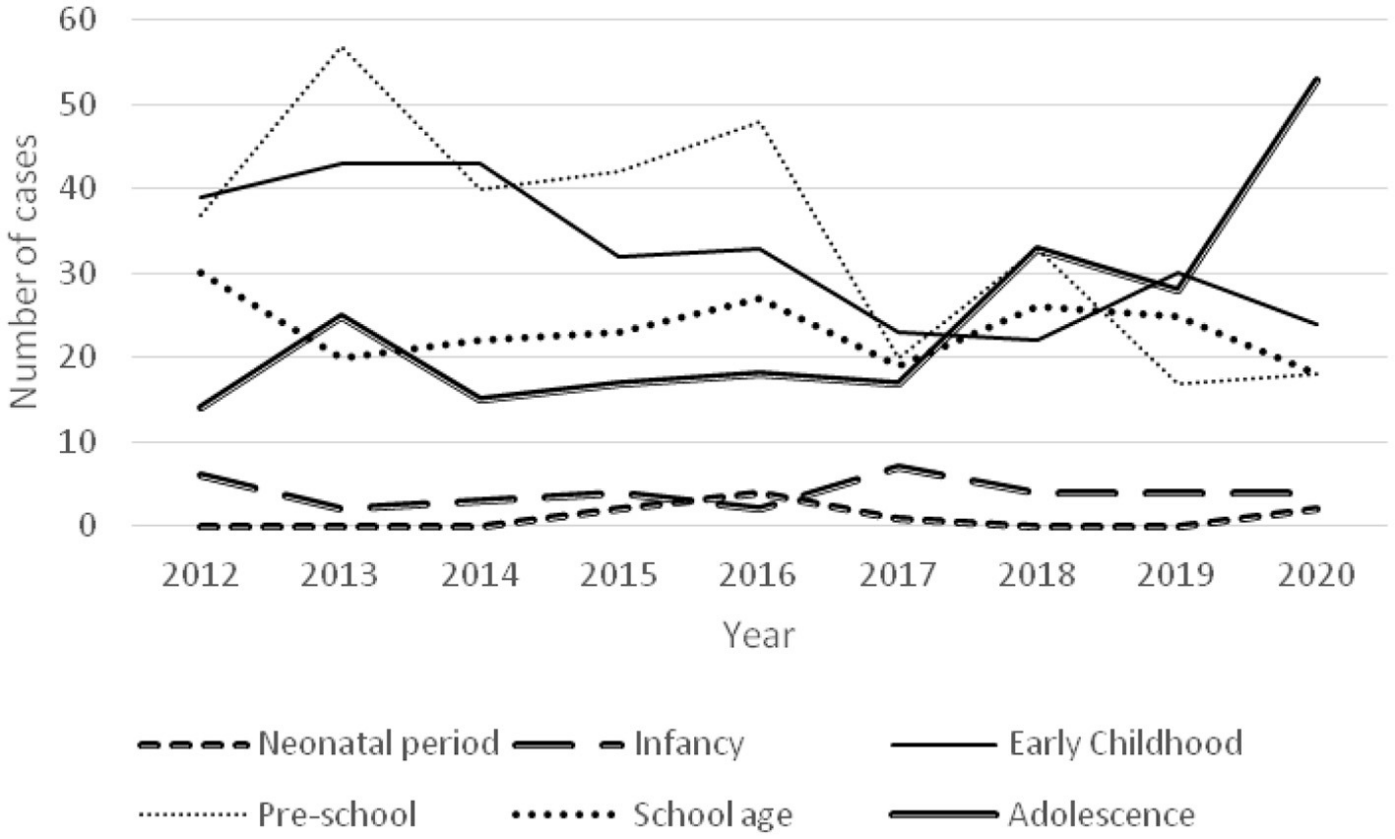
Time-varying curve of the number of children in each age group.

The age distribution of poisoning was not significantly different from 2012 to 2017, but the proportion of poisoning in adolescent children increased from 2018 to 2020 (Figure 3).

#### Poisonous Substances

**Figure 4:**
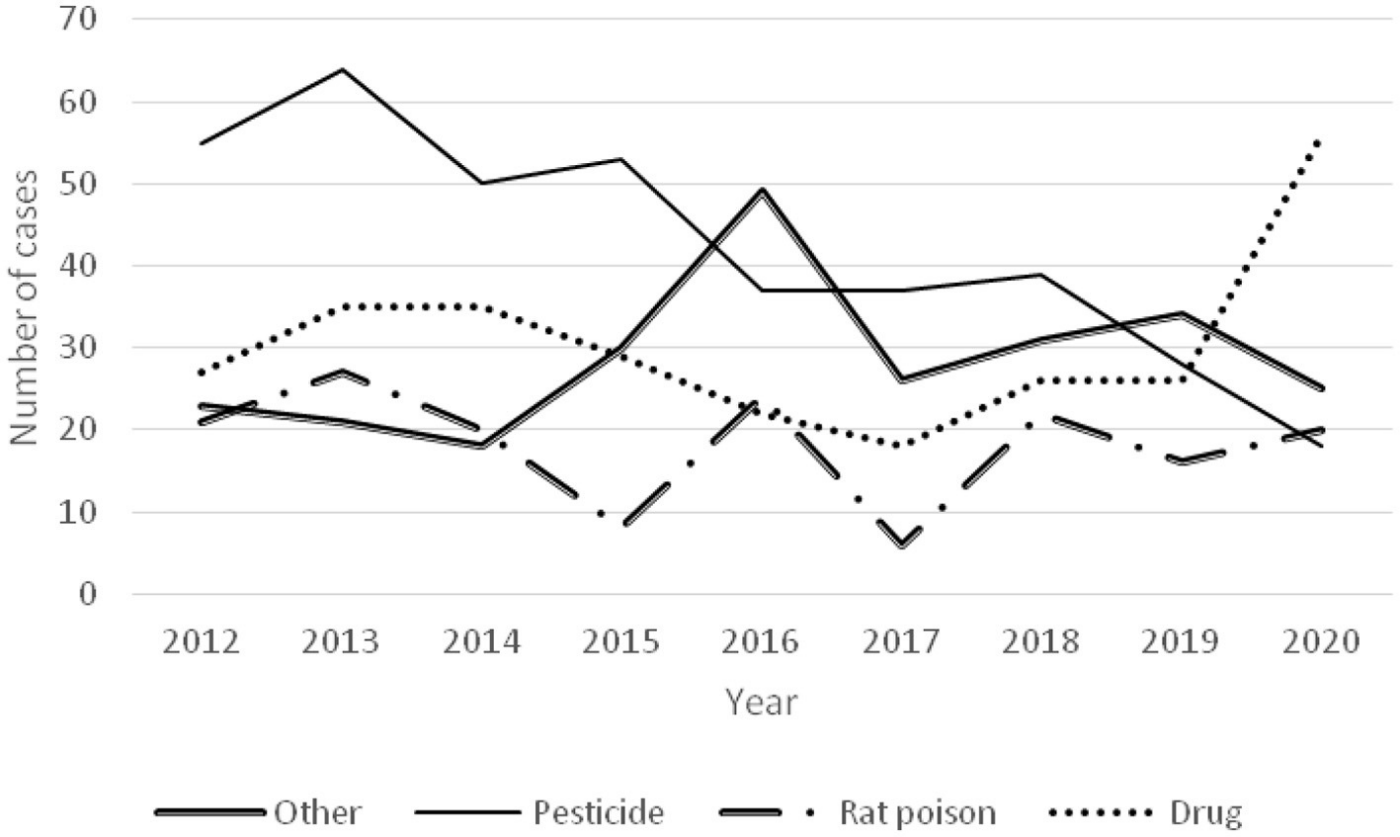
Time-varying curve of each poisoned substance and number of poisoned children in rural and urban areas.

**Figure 5:**
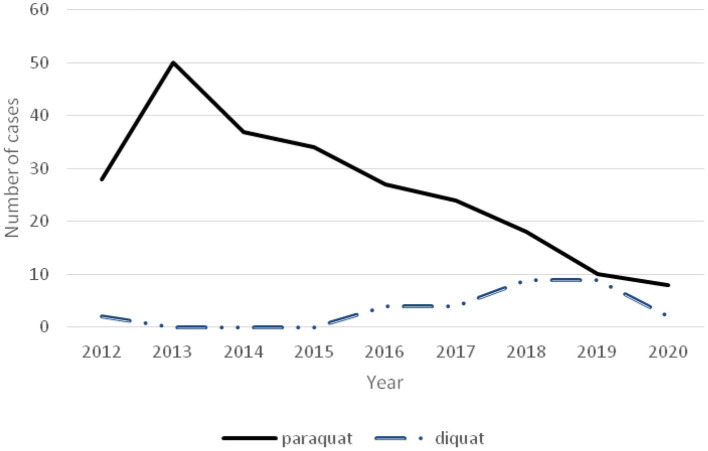
Time-varying curve of paraquat and diquat poisoning.

Pesticide poisoning showed a downward trend (Figure 4), accompanied by a decrease in the number of paraquat poisonings in recent years but an increasing number of diquat poisonings (Figure 5).

#### Causes of Poisoning

**Figure 6:**
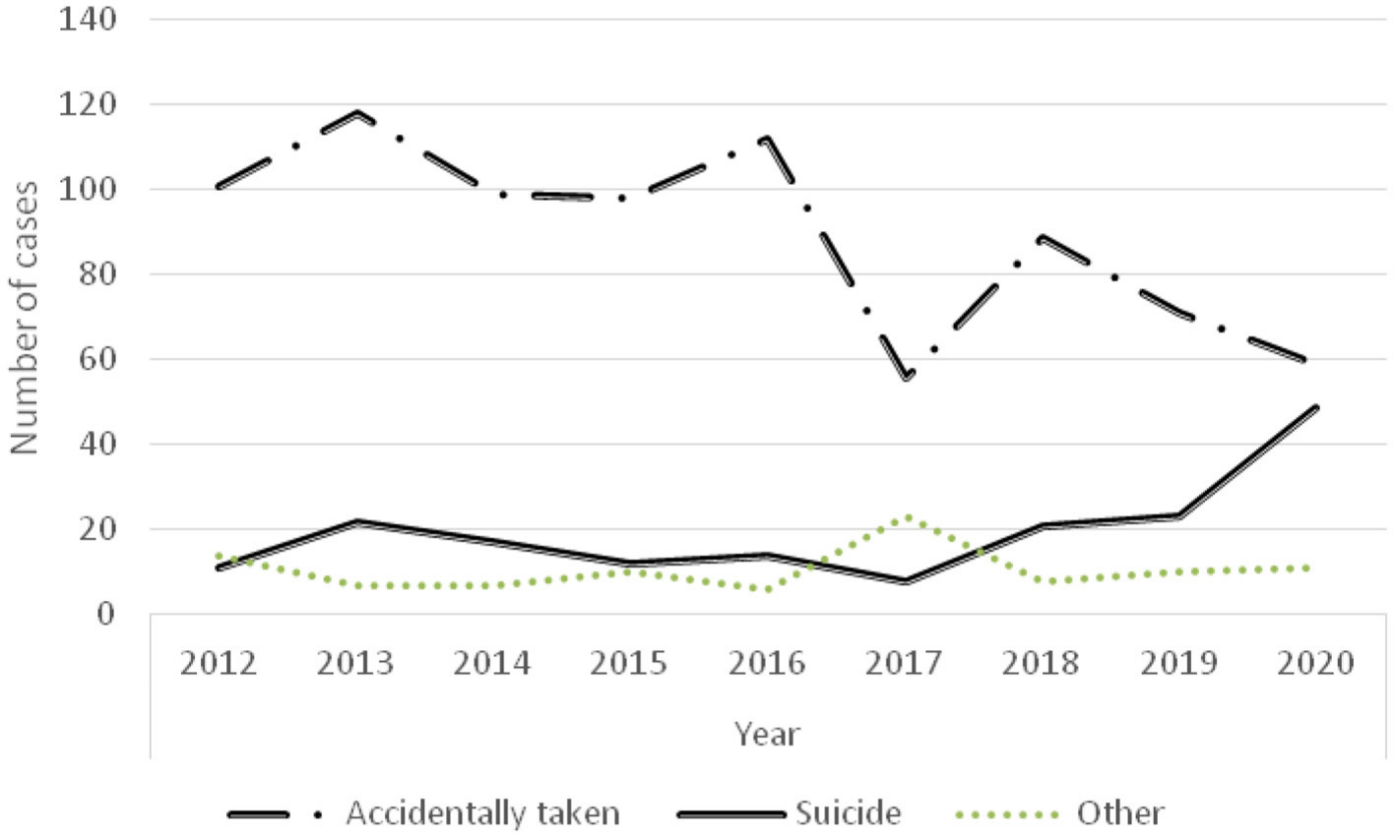
Time-varying curve of cause of poisoning.

As shown in Figure 6, the number of children with accidental poisoning declined, but the number of poisoning cases by suicide increased in the last 3 years (Figure 6).

## Discussion

### Gender Ratio and Age Structure of Hospitalized Children With Acute Poisoning

There were 588 males and 488 females in hospitalized children with acute poisoning. This result may not only be related to the fact that boys are generally more curious and active, but also may be due to the higher proportion of males in hospitalized children (6). The traditional Chinese custom of giving preference to sons over daughters results in Chinese families paying more attention to the health of boys, especially in the poor rural areas where families are more willing prioritize treating males (7).

The age structure results showed that infants and preschoolers accounted for the highest numbers of poisoning, which is generally consistent with the literature (5). Children in these age groups are generally curious but lack the ability to judge hazards (8). Our findings show that the number of poisoning cases in adolescent children has been increasing, and poisoning in adolescent children was significantly associated with suicide.

### Poisoning Caused by Pesticides, Drugs, and Other Products

The nature of substances causing acute poisoning in children varies in different countries and regions due to differences in national conditions, social status, and ethnicity. Drugs and household product poisoning are predominant in European and American countries (5). It has been documented that drug poisoning (chemical products and household cleaners) is most common in children aged 5 or younger, and there has been an increasing trend of opioids acute poisoning in children from 2005 to 2018 according to the US National Poison Data System (NPDS) (9). The Spanish Society of Pediatric Emergency Medicine Working Group on Poisoning evaluated 1,749 cases of poisoning children between 2008 and 2017, and the top three reported toxicants were drugs, household products, and alcohol (10). Drug poisoning is also the most predominant type of hospitalization for poisoning in aboriginal children in New South Wales (11). Acute poisoning in Iranian children aged 1–4 years old is predominantly drug-based (12). Pesticides are most commonly reported in developing agricultural countries such as India, China, South Africa, and Sri Lanka (13), which is consistent with the results of this study.

The number of poisoning cases was higher in children living in rural compared to urban areas. Pesticides have become the most common substance causing poisoning in rural areas, this is consistent with the relevant research results (4). Poisoning caused by herbicides and organophosphorus comprises the two major types of pesticide poisoning. Due to the ban of paraquat usage in China in 2016, the number of paraquat poisoning cases has sharply decreased. However, the number of poisoning caused by other types of herbicides has increased, such as diquat (which has a similar high toxicity and lethality rate as paraquat). Similar findings were reported in a Korean study (14). In addition, the number of cases of rat poisoning also increased. After the Chinese government explicitly banned the use of highly toxic rodenticides and promoted the use of anticoagulant rodenticides (bromadiolone and dalon), poisoning caused by anticoagulant rodenticides significantly increased compared with the previous ones. Reasons for poisoning could include poor economic and sanitary conditions in rural areas, low education of rural people, relatively weak safety awareness, and improper storage of pesticides. In our study, there were a few cases of children accidentally taking paraquat by pouring it into beverage bottles and storing it. There has also been an increase in the number of children in rural areas who lack parental care and supervision in recent years (15), which has greatly increased the risk of accidents. In several countries, it has been reported that there is a significant increase in accidental injuries among children who are cared for by someone other than their parents or those with low social status or level of education (13, 16–19). Moreover, Sri Lankan scholars found that the strongest risk factors for accidental poisoning of local children were improper supervision, working mothers, and improper storage of poisonous substances (13).

Accidental administration of drugs is the main cause of poisoning in children who live in towns or cities and have less chance of being exposed to pesticides. Our study showed that drug poisoning was predominantly caused by psychotropic, cardiovascular, and cold medicines. We also found that 72 children and/or families had chronic diseases requiring long-term drug use. These drugs can lead to accidental poisoning if they are placed where children can easily access them or if they are improperly administered.

Reducing poisoning from pesticides, drugs, and other products requires concerted efforts from many aspects. One aspect lies in strengthening regulation at the governmental and social levels. Others have reported serious poisoning cases caused by illegal sales of pesticides and drugs in small street stores (20–24). Therefore, relevant state departments should designate appropriate laws and regulations to emphasize design safety, control, and sales measures. For example, pesticide packaging should further emphasize its toxicity labeling, uses, and safe storage methods; food safety supervision should be strengthened to avoid the inflow of rat poison contaminated rice, noodles, and meat into the market; and “street pesticides” should be reduced by strengthening enforcement of illegal pesticides. However, there is limited evidence that environmental change interventions could reduce the risk of accidental poisoning in children (25, 26). Therefore, there is also a need to strengthen child safety awareness to avoid accidental poisoning.

With increased efforts to regulate highly toxic substances in China and improved medical care in primary care hospitals, the number of cases of hospitalized children who are poisoned has gradually decreased. The trend of changes in poisonous substances over the past 9 years showed that the number of children poisoned by pesticides decreased significantly and the number of children poisoned in rural areas also decreased. The reasons might be related to the strengthening of state regulation of pesticides and the improvement of people's knowledge about children's safety. The decreased number of children living in rural areas due to migration might also be contributing to the reduction of poisoning cases in rural areas (27).

### Suicide Poisoning

In this study, the majority of suicide cases were adolescents and females, and the number of suicide cases was higher in urban areas. Some suicide cases had a history of depression, and all children had life or study disputes with family members, classmates, or teachers before committing suicide. The number of suicide poisonings increased over the last 3 years, including an increased number of adolescent poisoning (Figure 6). Previous researches have shown that heavy burden from study is very common in adolescents committed suicide by taking poison (28). The suicide rates of children are higher in urban areas compared to rural areas, which might be attributed to greater study pressure of urban children originated from more family educational investment. While problems surrounding the mental health of left-behind children are prominent among the poisoning children in rural areas, and it is more common for left-behind children who are lack of care and love for a long time become to be introverted, lonely, insecure, and easily go to extremes (15). Poisoning suicides among adolescent girls might be associated with an awakening of their sense of self, unstable emotions, increased pressure from studies, society, and even sexual relations. Meanwhile, females are more emotionally rich and less resistant to frustration than males (29). Scholars in Taiwan have reported that female adolescents are also at high risk of poisoning suicide (30). In this study, one female case committed suicide by taking medicine because of the long-term sexual abuse from her stepfather. This example emphasizes the need for more awareness of child sexual abuse and protecting underage females.

Chronic mental illnesses such as depression are more prominent among poisoning cases in children with underlying diseases, and some foreign scholars have suggested that chronic physical and mental illnesses are associated with an increased risk of self-harm and suicidal ideation. Measures to assess and intervene with this population should be developed to effectively prevent these poisoning outcomes (31, 32).

There are some limitations in our study. We only included hospitalized children who have suffered from acute poisoning who were severely ill. More statistical studies are needed for children who are non-severely ill in the outpatient setting and who are not hospitalized. In addition, this is only a 9-year retrospective study with a minority of missing or inaccurate data. Children's Hospital of Chongqing Medical University is the largest national 3A pediatric hospital in southwest China, so the data in this study is regional representative but may be different with data from primary medical institutions (33, 34).

## Conclusions

Acute poisoning in children causes both familial and societal burdens. We call for state, school and family awareness of child poisoning to reduce risk of death and significant harm. First, we propose strengthening supervision at the governmental and social levels to reduce the unregulated circulation of pesticides and drugs. Secondly, we should reinforce the publicity of child safety knowledge and enhance the awareness of drug safety in families to protect children's health. Thirdly, there is an urgent call to focus on children's psychological safety, but there is a lack of professionals in child psychiatry in China who are qualified to treat the psychological defects of children with depression or suicide. In addition, the levels of medical care in primary hospitals vary, and the level of treatment for acute poisoning in children in some rural primary hospitals needs to be improved. These are key areas to address to reduce accidental poisoning in children in the future.

## Data Availability Statement

The original contributions presented in the study are included in the article/Supplementary Material, further inquiries can be directed to the corresponding author.

## Ethics Statement

The studies involving human participants were reviewed and approved by the Institutional Review Board of Children's Hospital, Chongqing Medical University. Written informed consent from the participants' legal guardian/next of kin was not required to participate in this study in accordance with the national legislation and the institutional requirements.

## Author Contributions

ZL and LT designed the study. ZL, LX, LY, SL, and LT collected and analyzed the data. ZL, LX, and LT drafted the manuscript. Critically revising the article was done by ZL, LX, and LT. All authors read and approved the final manuscript.

## Funding

This work was supported by 2019 Annual Philosophy and Social Science Projects of Chongqing Medical University (No. X9842).

## Conflict of Interest

The authors declare that the research was conducted in the absence of any commercial or financial relationships that could be construed as a potential conflict of interest.

## Publisher's Note

All claims expressed in this article are solely those of the authors and do not necessarily represent those of their affiliated organizations, or those of the publisher, the editors and the reviewers. Any product that may be evaluated in this article, or claim that may be made by its manufacturer, is not guaranteed or endorsed by the publisher.
